# Collaboration in providing intimate-partner violence services to women with disabilities

**DOI:** 10.1186/s12889-024-19352-6

**Published:** 2024-07-11

**Authors:** Fredinah Namatovu, Jens Ineland

**Affiliations:** 1 Department of Epidemiology and Global Health, Umeå University, Umeå, SE-901 87 Sweden; 2 Centre for Demographic and Ageing Research (CEDAR), Umeå University, Umeå, SE-901 87 Sweden; 3 Department Social Work, Umeå University, Umeå, SE-901 87 Sweden

**Keywords:** Collaboration, Disability, Disabled, Intimate partner, Violence, Support, Services

## Abstract

**Background:**

There is a consensus among scholars, policymakers, and implementers that addressing the complex nature of intimate partner violence (IPV) requires a collaborative response. However, there is limited literature on how various professionals work collaboratively to address the needs of women with disabilities who experience IPV. This study combines the perspectives of women with disabilities and those of professionals to understand collaboration in providing IPV services to women with disabilities.

**Methods:**

Twenty-nine in-depth interviews were conducted with 18 IPV service providers and 11 women with disabilities. The data were analyzed using reflective thematic analysis.

**Results:**

The findings are presented under three themes: the first shows a consensus among different IPV service providers and disabled women on the importance of collaboration when supporting victims of IPV with disabilities; the second depicts the common ways in which collaboration occurs when supporting women with disabilities; and the third illuminates the critical elements that boost effective collaboration.

**Conclusion:**

Supporting IPV victims with disabilities requires active collaboration at both an internal and external level. Strengthening collaboration among different actors requires trust, specified roles, and the allocation of adequate resources.

**Supplementary Information:**

The online version contains supplementary material available at 10.1186/s12889-024-19352-6.

## Background

Intimate partner violence (IPV) against women with disabilities is a complex societal and public health problem [1, 2]. According to the World Health Organization, approximately 15% of the world’s population is estimated to live with some form of disability, making it one of the largest minority groups in the world [3]. This number is expected to rise due to factors such as wars, natural disasters, and an overall increase in life expectancy. As people age, they may experience higher morbidity and disability rates, making it important to understand and address their health care needs [3].

In Europe, it is estimated that 16% of women live with a disability and that approximately 40% of these women have been subjected to violence at some point [4]. Further evidence suggests that women with disabilities are at increased risk of exposure to different types of violence, including IPV [1, 2, 5–10]. This finding was recently reaffirmed in a systematic review, which showed that the frequency and risk of IPV was greater among women with disabilities than among their non-disabled counterparts [11]. Women with disabilities experience violence from a range of perpetrators, including family members, service providers, personal assistants, and strangers, among others [11, 12]. Exposure to IPV among people with disabilities is exacerbated by intersecting vulnerabilities such as having a disability, being a woman, living in poverty, dependence on others for day-to-day support, and negative societal attitudes toward disability [11, 13].

Exposure to IPV is associated with numerous negative physical, emotional, and financial consequences that have an impact on health and well-being and therefore require a timely response [14, 15]. Given the pervasive nature of IPV, this response tends to come from multiple actors, which introduces complexities in accessing and delivering IPV services. Current research suggests that women with disabilities face pronounced disadvantages in accessing life-saving maternal healthcare and domestic violence services [1, 11]. Aside from access challenges that women face in general, women with disabilities face additional disability-related barriers increasing their vulnerability [3, 5]. Unfortunately, research and public health programs targeted at women with disabilities are inadvertently lacking [3, 4]. Given this background, there is a need for more research focusing on women with disabilities to increase our knowledge and understanding of their needs within the field of public health.

There is a consensus among scholars, policymakers and implementers that addressing complex societal problems requires collaboration among multiple actors [16, 17]. Collaboration is shown to effectively reduce the risk of IPV, ensure the safety of IPV victims, and hold perpetrators accountable [18]. Collaboration when responding to victims of IPV with disabilities is especially important given that certain disabilities pose additional accessibility challenges [19].

In this article, we consider collaboration to be a process that involves multiple actors working together [20]. The nature of collaboration tends to look different depending upon what systems are in place to provide IPV services. Sweden is a welfare state, thus, numerous IPV services are freely and centrally provided by the state. Systems that provide services within the welfare state generally respond well to complex problems due to interactions among cultural, social, economic, and political conditions [21]. However, an increasing number of target groups, together with the complexity of their needs, have contributed to a more differentiated welfare sector [21], leading to the fragmentation of services.

It is one thing to endorse ideas about professional collaboration, but it is quite another, and far more complicated, to administer, coordinate, and implement such collaborative activities in practice, and to achieve satisfactory results [22]. Some studies have noted that collaboration requires the establishment of a common goal, as well as having collective views, perceptions, and infrastructures for communication at different levels [21, 22]. However, it remains to be explored whether the same conditions promote effective collaboration when responding to the needs of disabled women subjected to IPV.

In Sweden, attention has been primarily directed toward how to coordinate and manage collaboration between IPV services at a practical level for women in general. To date, no research has investigated whether disabled women find collaboration helpful when they are accessing IPV services, or whether collaboration requires similar or different conditions when responding to the needs of women with disabilities. Given that the use of IPV services requires planned and coordinated contact with several agencies, including healthcare, social work, the police, and justice, this process can be complicated in the context of certain disabilities and therefore may require collaboration to have special considerations. At the same time, a differentiated welfare system with a fragmented structure of IPV service provision without functioning collaboration may pose unique challenges to women with disabilities.

The origin of our study is a systematic qualitative interrogation based on data collected from women with disabilities and IPV service providers in Sweden. Although there have been studies conducted elsewhere, to the best of our knowledge, this is the first study in Sweden to investigate collaboration in providing IPV support to women with disabilities from the perspectives of the women with disabilities themselves and IPV service providers.

## Aim

The specific aim of this study was to document the nature of collaboration while highlighting elements that encourage it in the provision of IPV support for women with disabilities.

## Methods

### Methodology

This qualitative study was based on in-depth interviews with IPV service providers and women with disabilities who had experienced IPV and had subsequently sought professional help. A constructivist epistemological lens was used, acknowledging that our scientific knowledge is shaped by our experiences and perceptions and that what we may regard as meaningful is often socially produced and reproduced through the interplay of subjective and intersubjective constructions [23]. We embrace reflexivity, maintaining that our own experiences and understandings of the world influence the research process [24].

### Study design

This study is part of a large, ongoing DIS-IPV project that seeks to evaluate different aspects of accessibility and utilization of IPV services by women with disabilities [25, 26]. We analyzed data from 29 in-depth interviews, of which 18 were with IPV service providers and 11 were with women with disabilities. Information about the study was disseminated through multiple channels, which included direct contact with professionals of interest. The women were mobilized through community-based organizations, and announcements were distributed through disability-related websites, disability organizations, membership magazines, social media, national radio, and women’s shelters. The recruitment period lasted for approximately 10 months.

The study participants gave informed consent to participate verbally and by providing a signature. Before the start of each interview, the participants were reminded of their right to withdraw their participation at any point, that they were free to respond only to questions they felt comfortable talking about, and that recording of the interview could be stopped upon their request. Although the original plan was to interview participants face to face, this changed due to the COVID-19 outbreak, and all the interviews were conducted digitally. The meetings with service providers lasted (on average) 60 min while the interviews with the women with disabilities lasted between 50 and 100 min.

### The participants

#### Women with disabilities

The majority of the interviewed women initiated contact with us, requesting to participate. This was often due to having seen the project announcements on the various channels, while a few learned about our project from the service providers with whom they were in contact. All the women self-identified as having a disability, having been previously subjected to IPV, and having been in contact with at least one IPV service provider. All participants were adults, aged between 25 and 60 years; five were employed part-time, while four had children from previous relationships. Several women reported having multiple disabilities. The most common forms of disability reported by the participants were mobility impairment, hearing impairment, eating disorders, personality disorders, schizophrenia, attention deficit hyperactivity disorder, stress-related disorders, post-traumatic stress disorder, depression, and anxiety disorders.

#### Service providers

The 18 service providers included in this study worked in healthcare, social work, the police, women’s shelters and the Center against Violence. Participants who worked within the healthcare service included physicians, physiotherapists, counsellors, and psychologists. Some of the social workers were employed by the local government, and some worked at women’s shelters and at the Center against Violence. The job descriptions of the social workers included counselling clients (with or without disabilities) who had experienced all forms of violence and abuse. Aside from counselling, the professionals working at the women’s shelters also offered support to women in need of safe houses, by securing them in secret locations where they could be protected from further abuse from their partners. The service providers working at the Center against Violence considered their primary task to be providing support for women and children who had been victims of IPV. The police officers interviewed in this study worked in violence units, and their specific role was to examine whether a violent crime has been committed. The interviewed service providers were at varying stages in their career paths, having worked for between two and 20 years.

### Interview content

Two open-ended interview guides were used, one for women with disabilities and the other for service providers. The questions in the interview guides covered a range of topics and were tailored differently to suit the two groups. The interview guide used for women with disabilities consisted of questions covering four broader categories: (a) accessibility and contact with IPV support and services; (b) quality of IPV support services and suitability; (c) competence development and recommendation, and (d) COVID-19 and IPV. A detailed list of the specific questions that were included in the interview guide has been previously published [26]. The interview guide for service providers covered the following broad topics; (1) Availability and establishing contact with support and assistance; (2) quality of available information and services; (3) barriers and facilitators to quality IPV services; (4) assessment of own competence. The specific questions included in the interview guide for service providers is included as supplement file 1. Follow-up questions were added during the interview sessions based on the participants’ narratives. We recorded all the interviews, which were later transcribed verbatim, and translated into English.

### Analysis

The data from the interviews were analyzed following the six-phase process of reflective thematic analysis [27–30]. The first phase of familiarization with the data involved listening to the audio recordings, and reading the written fieldnotes, memos, and transcripts. The transcripts were later exported into the MAXQDA data analysis software, where we developed codes and performed the first analysis. The second phase of analysis involved systematically reviewing the codes assigned to the texts and identifying codes with a shared meaning. During the third phase, we worked interactively and iteratively with the data, scrutinizing and grouping the codes to identify potential themes and subthemes. At this stage, we also identified quotes from the data that could potentially be used when writing the manuscript to illustrate the identified themes. Codes, potential themes, subthemes, and quotations were extracted from MAXQDA and exported to Excel for further analysis. The fourth analytical phase was performed in Excel and involved reviewing the potential themes and subthemes in relation to the coded data items and transcripts. This process involved constantly moving back and forth within the entire dataset and making further text comparisons. In phase five, we refined and named the themes, a process that resulted in three themes, which are presented in the results section, and in the final, sixth phase, we wrote the report. The data analysis process required moving back and forth, although here the analysis phases are presented chronologically [29].

## Results

We identified three overarching themes: (a) A shared view that collaboration was important; (b) Using collaborative spaces at internal and external levels; and (c) Pillars for achieving effective collaboration. The sections below offer a detailed presentation of these themes.

### Theme 1: a shared view on the importance of collaboration

This study revealed similarities between the views of professionals and women with disabilities on the role of collaboration in providing IPV services to women with disabilities. The two groups of participants viewed collaboration as central when addressing the complex and intersecting needs that arise when a victim of IPV has a disability. Nearly all the women in this study thought that collaboration helped to circumvent disability-related challenges. Experiencing IPV while having a disability expanded the magnitude of services a woman needed to seek. This complexity arises because disabled victims of IPV often need services from multiple providers. This process created an extra burden for the participants because most of the women were already in contact with other service providers due to their disabilities. Because of this broadened range of services, many women appreciated instances where service providers worked collaboratively to link them directly to other services through their ongoing collaboration, as opposed to leaving them to shoulder the task of navigating the process of seeking additional services on their own. In the example below, a participant reflects upon how collaboration helped her overcome disability-related barriers.

In the women’s shelter, they talked about having contact with the police, which I thought was good. That they had that contact…because for example, with my disability I have to run with papers here and there. I have to fill in things I kind of don’t know what they are, and I need a contact person to write something on a form. To do all this while dealing with my diagnoses [disability], I feel that’s a major shortcoming. (PWD 1)

This participant appreciated the fact that the women’s shelters worked in close contact with the police because this meant that she did not have to establish this contact on her own. The participant also noted that her disability made it difficult to contact all the multiple providers, and therefore working collaboratively made a large difference.

The professionals interviewed for this study reflected upon two dimensions in which collaboration was essential when responding to women with disabilities. The first dimension was related to providing support that is tailored to the unique needs of each disabled woman. The service providers noted that, in the context of disability, exposure to IPV creates unique needs that can best be addressed within a collaborative framework. Service providers with a well-established collaboration found it useful to engage these networks to work jointly to support disabled women. In this form of collaboration, service providers were already working in collaboration, but when they had a client with a disability, they met specifically to address the unique needs of each woman. This enabled them to provide services tailored to the specific needs of that woman.

If the person wants it, we’re happy to collaborate with other providers. We have a continuous collaboration…with the social services, the municipality, and the Center for Violence against Women. We meet them specifically around specific women…we do this if a woman wants to. Most of the time, she does want it because there’s a lot to keep track of. (AK, Women’s shelter)

As described by the service provider in the above quote, several service providers were aware of the complexity involved in accessing IPV services: “*There’s a lot to keep track of*”. Women needed to handle tasks such as filling in forms, keeping appointments, and making phone calls, among others. Such tasks, which are relatively common and often regarded as simple, could easily become overwhelming for women with certain disabilities. Another complexity that was discussed relates to the problem of the fragmentation of IPV services; as AK noted, the women needed to be in contact with different agencies. A similar experience was echoed by the women themselves, who listed the numerous actors they needed to contact, highlighting the challenge of navigating a fragmented IPV system. “*The social services is one*,* the family unit is another*,* it isn’t in the same building*,* but they recommend that you get help from these different people*” (PWD 10).

It was often difficult for the women to navigate IPV services that were provided by different actors situated in different locations, this was true especially for women with mobility impairments. Even when the services were provided by a single agency, it often required meeting on several occasions, a process described as “difficult” and “tiring” given a woman’s disability. This finding highlights the importance of professionals from different agencies working together through active collaboration to ensure that the disabled women receive all the IPV services they need, as opposed to merely providing information about what services are available or giving the women referrals without directly collaborating with other actors to ensure that they receive the support they need.

The second dimension in which service providers considered collaboration important was in relation to their own capacity-strengthening through information-sharing and learning from one another. Most of the interviewed professionals who had not received training on how to respond to women with disabilities who had been victims of IPV appreciated collaborating with others providers that had received such training and had shared their knowledge and experiences. Being part of a collaboration created new spaces for sharing experiences and learning from other professionals within the collaborative network. “*I personally think I’m helped by being part of some networks…meeting others who work on the same issue. It’s incredibly helpful. Then we also try to learn things*” (KN, Center for Violence against Women).

In the next example, HK describes how her unit collaborated with another agency to conduct joint training. Some felt that, more recently, the issue of IPV among women with disabilities is beginning to attract attention across various institutions, and this had opened up opportunities for collaboration and the exchange of knowledge and experience.

We invite social services to come and talk…I also know that the neurologists are working on this right now…in general they’ve raised this issue [intimate partner violence among people with disabilities]. (MK, Healthcare)

Another important issue that was raised was that collaboration enables the provision of long-term support for victims of IPV with disabilities. Initiating and maintaining long-term collaboration benefited the disabled women by enabling them to receive support from multiple agencies for an extended period of time. Long-term collaboration also guaranteed continuity of services provided by different actors, which helped to ensure that the women’s needs were not neglected over time. Even in cases where women terminated their use of IPV services, knowing that they could come back whenever they wanted was reassuring.

It feels important that you talk to each other in the network…sometimes women come back…we have recurring rehab periods with some…then you can also follow up…I think networking becomes very important. (KN, Center for Violence against Women).

In the example below, the service provider describes her feeling that support from her institution alone was not enough and explains that she had reached out to another institution.

She applied for an income support case…she came to visit me [at the office]. Then this man came along. And I figured there was no reason for him to sit in on visits between her and me. I felt that he was quite controlling, and what he said was a bit derogatory towards her…I connected her to the adult habilitation services and very quickly we got the adult habilitation involved. (RA, Social worker)

Collaboration was also considered important because it provided disabled women with a solid network that they could engage with for an extended period. Many victims of IPV with disabilities maintained long-term contact with the collaborative networks of service providers primarily because IPV occurred on multiple occasions. In some cases, the women needed to be supported for an extended period before IPV eventually stopped. Service providers felt that, for women who did not have stable networks of family and friends, receiving continuous support through collaboration was particularly important. Maintaining long-term contact with a network of professionals helped hesitant women to gradually make hard decisions such as separation from an abusive partner.

I felt that she needed more networking, especially because she didn’t have a network, the normal network with good stable parents and siblings and so on, so then we met together. (RA, Social worker)

In summary, the first theme illustrates mutual acceptance of the positive role of collaboration. In the second theme, we discuss how collaboration is organized.

### Theme 2: using collaborative spaces at internal and external levels

This study shows that collaboration in providing IPV services takes a variety of forms and functions, which we summarize as collaborative spaces that are internal and external. The external collaboration experienced by the service providers and disabled women involved instances of a provider working with other agencies outside their own, while in internal collaboration service providers primarily collaborated within the same organization.

There was a high level of agreement among service providers, regardless of their professional background, that external collaboration with various stakeholders was essential. The external collaborative approaches described by professionals involved working on larger structural issues and on issues that were specifically focused on IPV among women with disabilities. Collaboration on larger structural issues involved working together on central elements that affected the general operations of IPV services. For example, professionals described working together to influence political decisions, policies, and programs that affect the delivery of IPV services.

Externally, we collaborate at the right level with other authorities. To review preparatory agreements that the region will take a position on that govern the work of the unit…rewrite agreements…improve the working environment and patient safety for regional employees. Review guidelines, approved by policy and top official management. (SI, Healthcare)

From the women’s perspective, external collaboration was experienced mainly as the joint efforts made by multiple actors to provide services that meet the individual needs of women with disabilities. The disabled women appreciated instances where a service provider from one institution used their ongoing networks to aid women in accessing IPV services offered at another institution. This was accomplished in various ways; where disability made it difficult for the woman to articulate her needs, professionals would accompany her and help explain the problem to other actors. In the case of PWD 3, she had tried several times on her own to obtain help, without success; however, a breakthrough was achieved when a service provider actively collaborated with another actor:

I got to visit the health center…I had tried to apply for psychiatry myself…I’d been rejected, but things changed when I had a staff member with me on that doctor’s visit. I took that paper with me which said how bad I felt…then they could help me…this is much better. (PWD 3)

Through external collaboration, service providers were able to connect women to other IPV services they deemed necessary, even when the women were not in a position to recognize this need themselves. In the next example, this woman appreciated a police officer who not only referred her to the emergency room but also took the extra step of accompanying her.

I came through the emergency room myself actually. Or rather…in a way, it was the merit of the police…a pretty good police officer who looked at me and said “you don’t feel okay, so we’ll take you there. (PWD 5).

This woman had managed to go to the police station to report the abuse; however, she had not considered seeking medical help; it was the police officer who made this recommendation and accompanied her to the emergency facility.

Even though external collaboration was valued, some of our participants had not taken part in this form of activity. The service providers who had not engaged in external collaboration while providing IPV services to disabled women cited time constraints as the key limitation. Collaboration on every single case was viewed as time consuming, especially among service providers who did not regard IPV response as their primary task. Some professionals within primary healthcare felt that their principal task was providing medical care to patients and that women could access IPV services via other providers whose primary task was geared towards violence. “*What we know*,* the police are working on this. There’s also the women’s shelter. There are psychologists. We know that you can refer*,* but I haven’t referred*” (DW, Health worker). This provider had not referred any disabled patients to other IPV services because none of the patients had explicitly requested it.

Similarly, some women stated that they had not witnessed any form of collaboration between different institutions while seeking IPV services. In the example below, the woman had been in contact with several service providers, who merely recommended that she contact a women’s shelter, without becoming involved.

I didn’t get anything concrete, it was like, where should I turn? I had to look it up myself, but at the same time it was confusing because I didn’t know who to contact, which women’s shelter, there are several around the country…I had absolutely no idea, except that I could get in touch…thanks to social media and such self-help groups…who gave me tips. (PWD 5)

Internal collaboration took place within a specific agency, and often involved staff from different departments within the same institution working together on a specific case, or holding joint capacity-building sessions. “*We’re two curators who work here*,* and if we have difficult cases*,* we can supervise each other*” (MK, Health worker). If a provider felt that they needed extra support while handling a “difficult case,” she/he would invite another team member to join the session to offer support and supervise the process. Regarding capacity building, some described receiving regular training on different aspects of IPV matters, as MK explained: “*Yes*,* once a month we have group tutoring*,* along with others.*”

In summary, collaborating both internally within a given institution and externally with different institutions was considered important in supporting disabled women exposed to IPV, although it did not always work as expected. In the final empirical section, we address the conditions that enable collaboration to meet the expectations of both disabled women and the IPV service providers who work collaboratively to provide IPV services.

### Theme 3: pillars for achieving effective collaboration

This study identified trust, respect, specification of roles, clarification of threats, and adequate resources as the pillars that help to establish effective collaboration among professional IPV service providers working with disabled women.

Collaboration requires establishing trust among different actors. The process of building and establishing trust evolved over time through regular interactions and dialogues among the different actors, allowing those involved to get to know each other. In the example below RA explains how successful collaboration was established through regular interactions.

Some meet with those working at the women’s shelters together with other parties…to talk a little bit about what’s happened lately, what does it look like, what needs exist…Then we also have meetings with centers for violence against women, so that we kind of know who we are. When everyone knows who each other is, it’s always easier to interact. And then we’ve had big meetings with the whole IFO [the individual and family care administration unit]…and those working with probation and correctional services as well. (RA, Social worker)

Although the women considered collaboration to be important, many wished to be consulted before different service providers were included. Some women described instances when they had not trusted service providers who breached confidentiality by revealing the women’s personal information to other service providers without the women’s consent. Regardless of the importance of collaboration, many women felt that service providers needed to ask for their consent before involving other collaborators. In the example below, this participant felt betrayed by a professional who shared her details without her consent.

So, it turns out that he’s leaked some things to the university principal…I experienced this and, yes, it hurt. I trusted this person…I talked about my feelings. I’ve never talked about my emotions before; I’ve not been the [kind of] person who talks about emotions…When I talked, it felt safe, but then when I got to know everything else, there was no safety…I was really pissed. (PWD 1)

The service providers also highlighted the importance of seeking the disabled women’s consent before initiating collaboration with other actors. Collaboration was often not successful when service providers-initiated collaboration without involving women in making this decision or seeking their consent. This was viewed as a failure to respect the women’s autonomy which led to resentment of the IPV service providers. Disabled women were willing to forego IPV services when they felt that collaboration threatened their autonomy as exemplified by the service provider below.

One woman came to us here [at the adult rehabilitation center] she had been in contact with the psychiatry… her ex had handed over her contact details to his male friends who went to her house and rang the bell… She opened and they came in and raped her… After this incidence, she got a mental breakdown…and ended up in the psychiatric ward…There, then the doctor acted without contacting her, he called the police and made a report, contacted the social services office, and a lot of other organizations without this woman’s approval…This doctor was the one that also contacted us here…But then it so happened that this woman chose to discharge herself from psychiatry. She did not want to continue any contact with them. (ALG, counsellor)

The doctors’ decision to collaborate with other service providers was grounded in his professional responsibility to provide holistic care to patients. However, failure to engage the disabled women could also reflect professional attitudes towards people with psychiatric disabilities, as unable to articulate their needs and thus, incapable of making own decisions. Reclaiming her power back, this woman rejected collaboration initiated by professional, and opted to contact other providers on her own.

Finally, we noted that collaboration thrived in settings where service providers recognized and respected the work of other actors. In the example below, although RA’s institution offered support as requested by this woman, RA recognized that she needed a much more secure environment, which could only be provided by another institution.

Adult rehabilitation has a completely different opportunity to meet people, there the environment feels less threatening, at habilitation…they can meet people in many ways, me and the counselor, met and talked quite a lot. She [the counselor] was there [at the woman’s home] on a lot of home visits and it quickly became clear that he [the woman’s partner] was not nice to her, just as we had suspected. There was also physical abuse. They met at the habilitation center quite a lot and tried to talk about this…she separated from this man after many years…during this time…we got her an apartment and tried to support her in everything else…we applied for activity compensation [a financial allowance given to those with work disabling conditions]. (RA, Social worker)

This study also revealed that successful collaboration requires clarity around the roles and functions of the actors responding to the IPV needs of women with disabilities. Clarity about roles helps to avoid the duplication of services and to ensure that the needs of each woman are met, as explained by the service provider below.

It’s important to clarify as well because when there’s a lot of networking, I think then it’s important to clearly know who does what so no one thinks that’s what the others do…maybe you think they have contact with the counselor at the geriatrics, or they have contact with someone else…if you talk to each other, it becomes clear. (MK, Health worker)

Women with disabilities also shared a similar view on the importance of specifying the roles of the different actors engaged in collaboration. Clarifying roles helped to relieve anxiety and to offer reassurance that they had the right contact. PWD 10 recalled being confused as to whether she was in contact with the right person because the roles were not clearly defined.

I think someone from the women’s shelter was with me in the family court. But that’s not their job. I think it’s fuzzy, what are the tasks of the women’s shelter? What are the tasks of the family court?… I want this to be clearer, that if you end up here, you’ll get this help. (PWD 10)

The participants in this study observed that most sectors operated at the regional level, which created difficulties in sharing information between regions when women moved to other parts of the country. Even though IPV services are available nationwide, there are no standardized ways of sharing information across regions. Women felt that having national collaboration that allows the sharing of information would improve the delivery of services if a woman moves from one region to another. Currently, if a woman moves to another region, she has to re-start the process of seeking IPV services since there are no connections across regions. *“I wish that…there could be more coordination across the country.” (PWD 4).*

## Discussion

This qualitative study sought to understand the role and nature of collaboration in providing support and care to victims of IPV with disabilities through the lenses of women with disabilities and IPV service providers. The findings from this study indicate that the multifaceted needs of victims of IPV with disabilities require the problem-solving powers of multiple actors. Our findings further suggest that collaboration can occur either internally or externally to various institutions under the conditions of trust, respect, role specification, and adequate resource distribution.

The importance of collaboration in supporting and caring for victims of violence in the general population has been previously emphasized in violence research [16, 20, 31]. Our findings suggest that the need for collaboration might be more prominent when working with disabled victims of IPV due to the broader scope of needs that arise from both having a disability and experiencing IPV. Effective collaboration can reduce the complexity associated with simultaneously accessing IPV and disability services from multiple agencies. Effective collaboration helps by shifting the burden associated with navigating a complex IPV system from the woman to the IPV services [32]. Hence, collaboration is critical when addressing specific issues of accessibility to IPV services and other broader disability-specific issues [33].

In this study, the service providers who mainly worked in the social service sector appreciated working with other actors within the same organization. However, due to the complexity of IPV interacting with disability, there was a broader consensus on the need for external collaboration. Both service providers and disabled women called for external collaboration to be scaled up to the national level, which would enable the easy sharing of information regarding specific cases. Strengthening national collaboration in supporting IPV victims with disabilities could be more effective if it starts with the state agencies formulating policies that directly target the needs of women with disabilities. Having such specific policies in place might result in the production and implementation of actions within the collaborative frameworks of the implementers in the different institutions that provide IPV services.

From this study, it was evident that successful collaboration requires moving beyond one institution, to work externally with other institutions. Sweden is in a better position when collaboration might be arranged centrally because it is a social welfare state where intimate-partner violence services are publicly funded and freely available to all citizens through government institutions. This same structure could be used to reduce IPV service fragmentation through promoting collaboration that stretches across institutions and regions in the country. The targeted actors within would include institutions that primarily address IPV among women with disabilities, but also secondary actors, and other government administrative branches to address larger system issues. This finding is not only relevant to Sweden but also other countries where IPV services are publicly funded.

Our findings are in line with previous research, which has identified a lack of clarity and trust and inadequate resources as challenges to multiagency collaboration [34]. Maintaining respect within a collaboration serves to reassure all those involved of their valuable contribution, which further encourages continuity. This study has identified long-term collaboration as essential for ensuring continuity of service provision to disabled women, but that it requires a high degree of dedication by the collaborators over a long period of time.

Collaboration did not only benefit women with disabilities, but the professionals also perceived benefits. Such benefits included receiving support from colleagues, capacity-strengthening, and information-sharing on matters related to meeting the needs of women with disabilities. This finding supports previous evidence from a study in which 115 individuals working with disabled children were interviewed, where the professionals felt that working together with other agencies helped professional development [35].

IPV services providers who had been in a collaboration considered having good communication, regular meetings, and training workshops led to a positive professional experience. Other studies have shown that collaboration among healthcare providers helped to improve their interaction and communication [36]. Highlighting the benefits of collaboration, might be an incentive for professional collaboration.

This study further revealed that failure to respect women’s autonomy led to unsuccessful collaboration. It was evident that service providers ought to be transparent when initiating collaboration to avoid misinterpretation of their intentions or making women with disabilities to feel marginalized. This finding is in line with a recent systematic review that identified lack of transparency as one of the factors that limited access to health services among women with disabilities [36]. Inability to consult with disabled women before initiating collaboration erodes the good intentions of collaboration as it leaves the disabled women feeling ignored [37].

In conclusion, our study provides evidence of the importance of collaboration in the effective delivery of IPV services to women with disabilities. Such collaboration can be organized both internally and externally to various institutions. We recommend that a good collaborative climate foster trust, respect, and role specification and be equipped with adequate resources.

### Electronic supplementary material

Below is the link to the electronic supplementary material.

Supplementary Material 1

## Data Availability

The datasets generated and/or analyzed for the current study are not publicly available due to the restrictions from the ethical review board. Researchers were granted permission to use this data within this specific research project. However, this data can be made available to interested scholars upon reasonable request by contacting the corresponding author. Researchers requesting access to this data are also required to obtain ethical approval from the Swedish Central Ethical Review Board, its contact information is: registrator@etikprovning.se, telephone: +46104750800.
