# Supplementary material for: Collaboration in providing intimate-partner violence services to women with disabilities
Source: BMC Public Health. 2024 Jul 11;24:1863. doi: 10.1186/s12889-024-19352-6 (PMC11241963; doi:10.1186/s12889-024-19352-6)
Supplement: Supplementary file 1 — Supplementary Material 1 [file 12889_2024_19352_MOESM1_ESM.docx]

**Qualitative interview guide for service providers offering support to women with disabilities exposed to intimate partner violence**

**Introduction**

- Ask for background details: age, activities, employment and length of employment

**A. Availability and establishing contact with support and assistance**

1. Describe the types of interventions your organization offers to people who are victims of intimate partner violence (IPV).

2. How do people with disabilities exposed to IPV usually come into contact with your organization?

3. What kind of information do you (or your colleagues) routinely ask to determine if a person with a disability is at risk of being exposed to IPV?

4. In addition to the various initiatives you offer in your institution, what other types of support and interventions can be offered to people with disabilities who have been exposed to IPV?

5. Please tell us about the form of collaboration between the institution where you work and other institutions that offer support to victims of IPV with people with disabilities.

**B. Quality of available information and services**

1. Describe the procedures implemented by your organization when offering support to people with disabilities exposed to IPV.

2. What measures has your organization put in place to ensure that people with disabilities who have been victims of IPV have access to support?

3. Describe the type of training you have received at your current workplace to improve support for IPV victims with disability.

4. What form of support is offered internally by your workplace (e.g. training, further education, meetings, discussions) to improve support for people with disabilities exposed to IPV?

5. What is your opinion on the quality of the existing interventions offered to people with disabilities who experience IPV?

6. Do you think that your organization offers sufficient support for people with disabilities who have been subjected to IPV? What are the areas for further improvement?

7. Based on your experiences, how do people with disabilities exposed to IPV perceive the quality of the existing IPV services received after exposure?

**C. Barriers and facilitators for quality IPV services**

8. Which aspects, at the individual and organizational level, promote and hinder the quality of the support offered to victims of IPV with disabilities?

9. Describe a situation when you found it challenging to support a person with a disability who had been subjected to IPV. What made this situation challenging?

10. How can the quality of the services you offer be improved? Which authorities must be involved for these changes to materialize?

11. What would improve and facilitate your own and your organization's work with people who have disabilities exposed to IPV?

**D. Assessment of own competence**

12. How do you judge your competence to provide quality services to people with disabilities who experience IPV?

13. In what ways can your capacity be strengthened?

**Thank you for your participation.**
